# A Study of Paraganglioma Cases With Non-European Ancestry

**DOI:** 10.7759/cureus.27854

**Published:** 2022-08-10

**Authors:** Sadia Ejaz, Neeharika Nandam, Susan Maygarden, Maya Styner

**Affiliations:** 1 Endocrinology, Diabetes and Metabolism, University of North Carolina at Chapel Hill School of Medicine, Chapel Hill, USA; 2 Internal Medicine, University of North Carolina at Chapel Hill School of Medicine, Chapel Hill, USA; 3 Pathology and Laboratory Medicine, University of North Carolina at Chapel Hill School of Medicine, Chapel Hill, USA

**Keywords:** genetic risk, precision medicine, african ancestry, pheochromocytoma, paraganglioma

## Abstract

Capable of generating excess catecholamines, untreated extra-adrenal paragangliomas (PGLs) result in severe cardiovascular morbidity and mortality. Increasingly, a hereditary basis can be identified to underlie PGLs, though such data are largely absent in populations of non-European descent. We present two patients with PGL, both exhibiting similar age, sex, and geographic ancestry. Our patients are unrelated, Kinyarwanda-speaking females from the Democratic Republic of the Congo. The first patient presented with lower extremity edema and poorly controlled hypertension and was found to have multifocal PGL in the abdomen and bladder, proven by biopsy and treated with surgical excision. Our second patient presented with palpitations, shortness of breath, headache, and hypertension, was found to have mediastinal PGL, and underwent surgical excision. Genetic testing was negative in both cases. The first patient has not shown recurrence based on active surveillance with imaging and biochemical testing. There is a concern for recurrence in the second patient, eight years after diagnosis, which is currently being investigated. Our second patient lived at a high altitude for most of her life, pointing toward a possible role of hypoxia in the pathogenesis of her tumor development. Our cases raise questions that require active inquiry regarding additional environmental and/or genetic factors that might predispose to PGLs in uncommon anatomic sites and in understudied, vulnerable populations.

## Introduction

Forward regarding ancestry

Genome-wide association studies (GWAS) are estimated to encompass a population of less than 2% of African ancestry [1]. Race does not fully capture human genetic variation, and vast differences are noted between Africans [2-6] Geographic distance, on the other hand, better describes such variations. Country of origin and language are referenced herein; however, this does not imply a particular ethnicity, as ethnicity does not necessarily correlate with language in this region of Africa. Kinyarwanda is one of approximately 550 unique Bantu languages [7] and the national language of Rwanda, but it is also spoken by citizens of eastern Congo, Tanzania, Uganda, and Burundi [8]. The patient in case one also referred to her language as both Kinyamulenge and Kinyarwanda interchangeably, though the former encompasses other dialects. Additionally, both patients have some knowledge of Swahili, and the patient in case two speaks some French. By specifying country and language, we apply ways in which our patients choose to self-identify. While we highlight differences, we aim to improve care for individuals whose disease might be understudied or poorly understood, hopefully navigating from race-based to *race-conscious *medicine [9].

Introduction to the cases

Adrenal pheochromocytomas (PCCs) and the less common extra-adrenal paragangliomas (PGLs) are notorious for a puzzling presentation and mimicry of many disorders [10]. Though most show some degree of hypertension, which can be paroxysmal, as well as orthostasis [10], diagnosis is notoriously delayed as highlighted by US President Eisenhower, who was diagnosed with PCC posthumously [11]. Autopsy and imaging studies also suggest that PCC/PGL under-diagnosis persists compared to actual disease numbers [12,13] and that even so-called “silent” PCC/PGL might cause harm [14-16]. Even with improved diagnostic methods for PCC/PGL, diagnostic delays persist, which might also be attributed to being from a marginalized group with less access to care. The natural history of untreated PGL includes the risk of devastating complications such as cardiomyopathy, myocardial infarction, myocarditis, pulmonary as well as cerebral hemorrhage, and pulmonary edema [17]. Thus, diagnosing PCC/PGL is of critical importance.

Structurally, PCC/PGL tumors represent chromaffin cell neoplasms, derived from neuroendocrine tissue, which have the capacity to secrete excess catecholamines [18]. Studies have shown that those with bilateral, multifocal disease, or early age at diagnosis, are at higher risk of having a causal spontaneous or inherited pathogenic variant mediating PCC/PGL [18]. PGLs arise from parasympathetic nervous tissue along the aortic arch, neck, and skull base, as well as sympathetic nervous tissue in the retroperitoneum, thorax, and other uncommon sites. The risk of metastatic disease is greater with sympathetic extra-adrenal PGL than with PCC [19]. To date, several PCC/PGL syndromes have been described including pathogenic variants in the following genes: *MAX*, *NF1*, *RET*, *SDHA*, *SDHB*, *SDHC*, *SDHD*, *VHL*, and *TMEM127* [20]. Prior large-scale analyses performed in European cohorts noted that at least 30% of this population’s PGLs possess germline mutations [18,21]. Based on these findings, practice guidelines from the Endocrine Society and other major organizations recommend universal genetic testing in PGL [21,22].

With evolving interest in precision medicine and its potential diagnostic and therapeutic benefits [23], finding new genes that underlie PCC/PGL has become increasingly important. However, such data are largely absent in populations of non-European ancestry. We present two unrelated cases of similar age and sex, both of African descent, with PGLs in anatomically unique sites. Both had norepinephrine (NE)-secreting tumors and tested negative for known PCC/PGL pathogenic variants. These cases raise questions about the pathogenesis and clinical presentation of PCC/PGL in understudied minority populations, as well as genetic and environmental factors that might contribute to tumoral pathogenesis.

## Case presentation

Case one

A 43-year-old Kinyarwanda-speaking woman, originally from the Democratic Republic of the Congo (DRC), presented to her primary care physician’s clinic with left lower extremity edema. Approximately 10 years prior, she relocated to Kenya, where she lived for five years before once again relocating to the state of North Carolina, USA. Along with edema, she was noted to be hypertensive at 154/86 mmHg and initiated on therapy with spironolactone-HCTZ 25-25 mg and furosemide 40 mg daily. Diuretics were prescribed to alleviate lower extremity edema and improve hypertension. Lisinopril 20 mg was added, and blood pressure improved to 123/70 mmHg. Her team began a workup with lower extremity Doppler ultrasound which was negative for thrombosis. Abdominal computed tomography (CT) revealed a 3 cm necrotic mass inferior to the duodenum, abutting the inferior vena cava and aorta, as well as two bladder wall lesions. As PGL was not yet considered in the differential diagnosis, she underwent endoscopic ultrasound-guided fine-needle biopsy of the inferior duodenal lesion, without alpha blockade or requisite hydration. Thankfully, she maintained hemodynamic stability peri- and postoperatively. Biopsy revealed dyshesive, bland cells with round-to-oval nuclei and anisonucleosis. Importantly, if there is suspicion for PCC/PGL, biopsies should be avoided due to high associated morbidity and mortality [24]. Immunohistochemical staining was positive for synaptophysin, chromogranin, GATA-3, and CD-56, while Keratin stain was negative. These results were consistent with a multifocal paraganglioma, as opposed to other neuroendocrine-derived lesions.

Labs revealed elevated plasma-free normetanephrines (NM) of 253 (0-145 pg/mL), while plasma-free metanephrine was <10 (0-62 pg/mL). Urinary 24-hour NE was elevated at 185 (15-80 µg). Urinary 24-hour NM was similarly high at 1,404 (119-451 µg; hypertensive <900) (Table 1).

**Table 1:** Clinical characteristics prior to and after PGL tumor resection in case one and case two. NM: normetanephrine; M: metanephrine; NE: norepinephrine, E: epinephrine; VMA: vanillylmandelic acid; TSH: thyroid-stimulating hormone; CgA: chromogranin A; MIBG: metaiodobenzylguanidine, ^18^F-FDG-PET/CT: fluorodeoxyglucose-positron emission tomography/computed tomography; GeneDx panel PCC/PGL: variants including *FH*, *MAX*, *MEN2*, *NF1*, *RET*, *SDHA*, *SDHAF2*, *SDHB*, *SDHC*, *SDHD*, *TMEM127*, and *VHL*; Invitae Hereditary PCC/PGL: variants including *MAX*, *NF1*, *RET*, *SDHA*, *SDHAF2*, *SDHB*, *SDHC*, *SDHD*, *TMEM127*, and *VHL* genes.

Test	Case 1		Reference Range	Test	Case 2		Reference range
	Before surgery	After surgery			Before surgery	After surgery	
Plasma-free fractionated NM (pg/mL)	253 (H)	98	0–145	Plasma-free fractionated NM (nmol/L)	7.1 (H)	0.55	<0.90
Plasma-free fractionated M (pg/mL)	<10	18	0–62	Plasma-free fractionated (M nmol/L)	0.22	0.25	<0.50
Urine NE (µg/24 hour)	184 (H)	18	15–80	Urine NE (µg/24 hour)		16	15-80
Urine E (µg/24 hour)	<1.7	<1.1	<21	Urine E (µg/24 hour)		2.4	<21
Urine dopamine (µg/24 hour)	285	20 (L)	65–400	Urine dopamine (µg/24 hour)		145	65-400
Urine M (µg/24 hour)	94	88	30–180 (normotensive) <400 (hypertensive)	Urine M (µg/24 hour)		101	30–180 (mormotensive) <400 (hypertensive)
Urine NM (µg/24 hour)	1404 (H)	297	119–451 (normotensive) <900 (hypertensive)	Urine NM (µg/24 hour)		224	119–451 (normotensive) <900 (hypertensive)
Urine VMA (mg/24 hour)	5.9	3.7	<8	Urine VMA (mg/24 hour)		2.6	<8
TSH (µIU/mL)	1.19	1.36	0.34–5.66	TSH (µIU/mL)	1.84		
CgA (ng/mL)			<93		265	64	<93
Genetic testing		GeneDx PCC/PGL Panel: Neg				Invitae Hereditary PCC/PGL Panel: Negative	
Imaging studies	CT abdomen/pelvis with contrast: necrotic 3 cm mass likely from duodenum and less likely the pancreas, enhancing nodules in the bladder wall, prominent uterus and cervix	CT abdomen/pelvis with contrast: interval post-surgical findings from TAH/BSO, bilateral pelvic lymph node dissection, cystectomy with neobladder formation			CT chest with contrast: large heterogeneously enhancing mass beneath carina and posterior to the left atrium		
	Whole body MIBG: MIBG avid aortocaval mass located just inferior to the uncinate process of the pancreas	Whole-body MIBG: there is no scintigraphic evidence of residual or recurrent MIBG-avid tumor			^18^F- FDG- PET/CT: posterior mediastinal mass with marked FDG utilization with a maximum SUV of 37.5		
					Whole-body MIBG: increased radiotracer uptake within the known middle mediastinal mass		

Extent of disease was localized via ^123^I metaiodobenzylguanidine (MIBG), showing avidity of the necrotic- appearing mass, inferior to the duodenum, which was noted previously on CT. Though Endocrine Society guidelines recommend imaging with ^123^I MIBG when therapy via ^131^I MIBG is planned, they also state ^123^I MIBG can be useful in those with an increased risk for metastatic disease due to the size of the tumor or extra-adrenal, multifocal disease [25]. Importantly, ^123^I MIBG is more sensitive than^ 131^I MIBG for PGL detection, and its utility increases when used in conjunction with single-photon emission computed tomography (SPECT) imaging.

**Figure 1:**
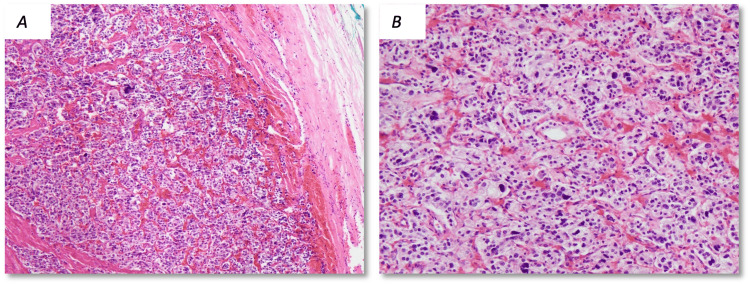
Case one pathology from bladder resection. Sections of the bladder resection from Case one show a neoplasm composed of lightly eosinophilic epithelioid cells with moderate nuclear pleomorphism with a nested arrangement (termed “zellenballen” architecture). The epithelioid cells were positive for immunostains for neuroendocrine markers (synaptophysin, chromogranin, and CD56). Between the cell nests flattened sustentacular cells can be found which stained for GATA-3. Both populations of cells were negative for pankeratin immunostain. This mass was largely encapsulated without invasion into surrounding tissues (hematoxylin and eosin stain; A: 40× magnification, B: 400× magnification).

The previously visualized bladder wall lesions were not MIBG avid. Cystoscopy revealed subepithelial intramural bladder lesions, with the largest measuring 2 cm in the largest dimension at the dome, as well as three posterior nodules measuring 5-10 mm each. Findings were inconsistent with urothelial carcinoma, and thus it was suspected that these bladder lesions represented extra-adrenal PGL. Transurethral resection of the bladder tumor confirmed a diagnosis of PGL. Peripheral blood leukocyte testing for PCC/PGL pathogenic variants including *FH*, *MAX*, *MEN2*, *NF1*, *RET*, *SDHA*, *SDHAF2*, *SDHB*, *SDHC*, *SDHD*, *TMEM127*, and *VHL* genes was negative (GeneDx, Gaithersburg, MD, USA). In addition to optimizing blood pressure preoperatively with atenolol, doxazosin, and lisinopril, the patient was placed on continuous intravenous hydration for 14 hours prior to surgical excision. At diagnosis, due to multifocality of the bladder lesions, as well as necrotic area near the duodenum, and a “prominent appearance” of the cervix/uterus on CT, there was concern for metastatic disease. This factored in the decision-making offering a full resection of the bladder, uterus, and ovaries. Thus, the patient underwent cystectomy with neobladder creation, hysterectomy, and bilateral salpingoophorectomy. Surgical pathology showed PGL with extracapsular and vascular invasion (Figure 1).

Postoperatively, urinary 24-hour NE and NM both normalized at 18 µg and 297 µg, respectively. Antihypertensives were successfully discontinued. Although pathology noted margins of the three bladder wall foci as negative, the retroperitoneal lesion demonstrated extracapsular extension and vascular invasion. Previously, MIBG was able to localize disease, and due to our limited understanding of PGL in these anatomic sites and the negative genetic testing, functional imaging in addition to biochemical testing was advised. One year later, MIBG/SPECT and CT of the abdomen were negative for recurrence.

Case two

**Figure 2:**
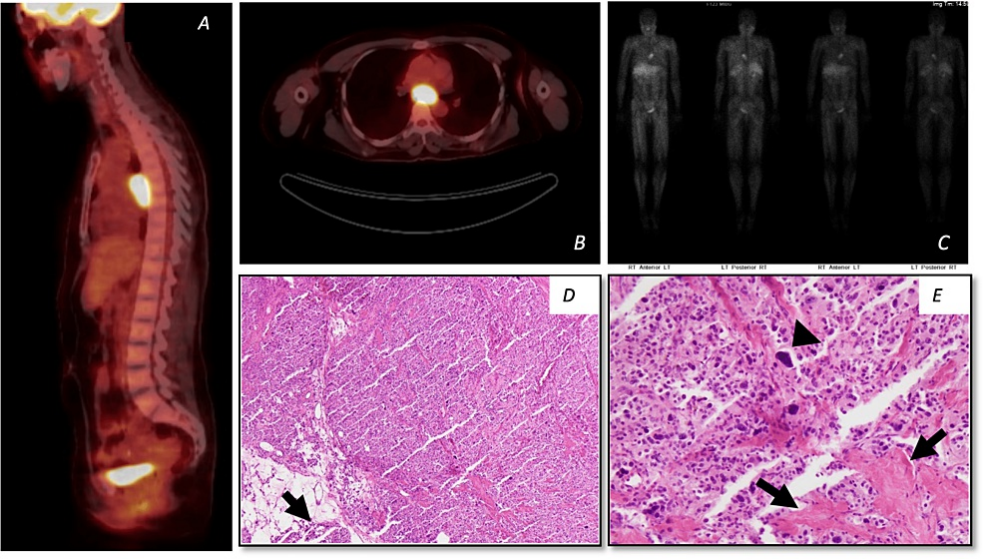
Representative imaging and histology from case two with mediastinal paraganglioma. Top panel (A) and (B) CT/FDG-PET showing posterior mediastinal mass demonstrating marked FDG utilization with a maximum SUV of 37.5. (C) MIBG showing increased radiotracer uptake within the known middle mediastinal mass. Bottom panel (D) and (E): Histologic features of case two. This neoplasm has similar underlying architecture to case one, with eosinophilic epithelioid cells with nested architecture. Immunostains were also similar, with the lesional cells positive for neuroendocrine markers (chromogranin, CD56, synaptophysin, and neuron-specific enolase) and the sustentacular cells positive for S-100. Both populations were negative for keratin stains. However, (D) shows that the tumor is not encapsulated and extends into the surrounding adipose tissue (arrow), and (E) shows very marked nuclear pleomorphism (arrowheads) and necrosis (arrows) (hematoxylin and eosin stain; D: 40×, E: 400×). While only metastases are definitive for a diagnosis of malignancy in paraganglioma, histologic features that are associated with malignancy include elevated mitotic rate, atypical mitoses, necrosis, spindling of tumor cells, capsular invasion, vascular invasion, and marked nuclear pleomorphism. This case demonstrates some of those features. Of note, it is case two who has some clinical concern, eight years after diagnosis, for recurrence, with a new suspicious lesion in the right kidney. CT: computed tomography; FDG: fluorodeoxyglucose; MIBG: ^123^I metaiodobenzylguanidine PET: positron emission tomography; SUV: standardized uptake value

A 41-year-old Kinyarwanda-speaking woman, originally from the DRC, presented with palpitations, shortness of breath, headaches, and hypertension. Ten years prior to her presentation, she relocated to Rwanda and soon after that to the state of Maine, USA and five years later to the state of North Carolina, USA. She presented with palpitations, shortness of breath, headaches, and hypertension. Her symptoms were episodic, lasting from ten minutes to two hours. Her only medication was ibuprofen for headaches, as needed, and she had limited consistent access to healthcare before her arrival in the United States three years prior. Medical history revealed hypertension diagnosed 15 years prior and intermittent use of antihypertensives, including an episode of hypertensive emergency for which she required hospitalization, and a 15-year history of difficulty to achieve fertility. Obstetric history was significant for several first trimester miscarriages and three term pregnancies. Her second full-term pregnancy was complicated by pre-eclampsia. The patient is one of multiple siblings; however, she denied a history of cancer or medical problems in the siblings. Interestingly, the only known family history was that of chronic headaches in the patient’s mother, referred to as migraines, which might well be relevant for this case, though the patient knew of no other afflictions, and denied a family history of malignancies, early stroke, or adrenal problems. Blood pressure was high at 182/118 mmHg and thus HCTZ 25 mg and labetalol 200 mg twice daily were initiated. An echocardiogram showed a 4 cm mass posterior to the left atrium. Chest CT showed a heterogeneously enhancing tumor beneath the carina and posterior to the left atrium. Fluorodeoxyglucose-positron emission tomography (^18^F-FDG-PET) displayed avidity with a maximum standardized uptake value of 37.5, consistent with malignancy (Figure 2, Panel A-C).

Endoscopic bronchial ultrasound and video-assisted thoracoscopy were performed, yet the tumor’s vascularity precluded an endoscopic biopsy. A surgical biopsy of the mediastinal mass was performed after thoracotomy, revealing marked cytologic atypia with high-grade nuclear features, multinucleated giant cells, and positivity for chromogranin, CD56, synaptophysin, neuron-specific enolase, and S100 consistent with extra-adrenal PGL (Figure, Panel D-E). This tumor also stained for the GATA-3 transcription factor, which in the literature has been noted to co-localize with S100 in cells that surround nests of PGL tumors [19,25]. Keratin staining was negative.

Plasma-free fractionated metanephrine level was normal at 0.22 (<0.5 nmol/L), while NMs were elevated at 7.1 (<0.90 nmol/L), providing biochemical confirmation of PGL. In addition to the prior antihypertensive regimen of HCTZ and labetalol, alpha blockade was initiated with doxazosin 2 mg twice daily, more than one month preoperatively with a good response: blood pressure recorded at 114/74 mmHg, heart rate 71 beats per minute several days prior to surgery. Resection of the mediastinal mass was performed; surgical pathology was consistent with PGL. Peripheral blood leukocytes tested negative for known *SDHB* variants (Mayo Clinic, Rochester, MN, USA). Additional testing for known PCC/PGL variants, including *MAX*, *NF1*, *RET*, *SDHA*, *SDHAF2*, *SDHB*, *SDHC*, *SDHD*, *TMEM127*, and *VHL*, was negative (Invitae, San Francisco, CA, USA). Hypertension improved postoperatively, and antihypertensives were successfully stopped. Follow-up plasma NM was 0.55 (<0.90 nmol/L) at one-year postoperatively and was normal for six years of follow-up, though eight years after the diagnosis, blood pressure is rising with a slight rise in plasma NM 0.70 (<0.90 nmol/L) and urine NE is higher than prior at 25 µg/24 hour. Along with a 2.6 cm right upper pole renal lesion, positive on MIBG, there is a concern for recurrent PGL. The patient is continuing to undergo biochemical and other investigations at the present time.

## Discussion

PCC/PGL is thought to carry the highest degree of heritability in human neoplasms, but many tumors remain genetically undefined [26]. In the past 20 years, pathogenic variants in 12 susceptibility genes have been reported, and research has demonstrated that more than a third of patients of European ancestry carry a variant in one of the following 12 genes: *NF1*,* RET*,* VHL*,* SDHD*,* SDHC*,* SDHB*,* SDHAF2*,* SDHA*,* TMEM127*,* MAX*,* EPAS1*, and *FH* [13]. Identification of new variants associated with PCC/PGL will serve as a powerful tool in identifying co-occurring cancers in hereditary syndromes and screening first-degree relatives. Recent work confirmed variants in novel genes such as *IDH *(*isocitrate dehydrogenase 1*), *PHD1* and *PHD2* (*hypoxia-inducible factor prolyl 4-hydroxylase 1 and 2*), *MDH2* (*mitochondrial malate dehydrogenase 2*), and *KIF1β* (*kinesin family member 1β*) [27], showing that much remains to be investigated for the molecular underpinnings of PCC/PGL.

Due to the heterogeneity and complexity of the disease, PCC/PGL tumorigenesis remains poorly understood in a sizable fraction of patients. Previous studies established that hereditary PCC/PGLs demonstrate distinct gene expression profiles correlating with biochemical secretory phenotype; for example, *VHL *is predominantly noradrenergic and *MEN2 *is mostly adrenergic [15]. The Cancer Genome Atlas (TCGA) molecular taxonomy divides these patients into the following three disease clusters based on tumor pathogenesis: (1) pseudohypoxia pathway, (2) Wnt-signaling, and (3) kinase-signaling. Each cluster’s unique molecular-clinical-biochemical-imaging phenotype can be applied to personalize care with precision medicine and targeted therapies [23].

Increased incidence of PGL in humans living at remarkably high altitudes was first described in 1976 by Arias-Stella et al. in a study of patients living in the Andean mountains, 4,000 m above sea level [28]. Their findings were supported by Baysal et al. in 2000, establishing hypoxia as a proliferative factor for carotid body chief cells [29]. The observation that the main oxygen sensing organ, the carotid body, could give rise to tumors under conditions of low oxygen was an indication that oxygen may act as an environmental modifier of tumorigenesis. Other studies have since proven chronic hypoxia as a risk factor for sporadic PGL [30]. Normoxic conditions facilitate the degradation of hypoxia-induced factor (HIF-1) via hydroxylation by oxygen-dependent prolyl hydroxylases (PHDs) and targeting of a VHL-E3 ubiquitin-protein ligase degradation complex. In the setting of low oxygen levels, HIF-1 protein escapes degradation and translocates to the nucleus, upregulating the transcription of genes that promote tumor growth [31]. Interestingly, high-altitude PGL has a greater than 80% female predominance [32]. Our second patient lived on the Mitumba mountain at an elevation of 2,990 m in south Kivu in DRC. As such, our cases contribute to prior literature about an association between high altitude and PGL, highlighting the need to study the role of hypoxia as well as other environmental and genetic factors in mediating extra-adrenal PGL.

We present two cases of extra-adrenal PGL, both showing not only similar age, sex, and geographic ancestry but also both negative for previously characterized causal variants from populations of European descent. Both had tumors at uncommon anatomic sites. Less than 10% of PGLs were previously thought to involve the mediastinum or bladder [33]. However, studies on the clinical characteristics and causative variants of PGLs in patients of non-European ancestry, especially those of African descent, are overall limited. Interestingly, the few studies that have examined PGL in patients from the African continent have described unique characteristics including a female predominance and a tendency for unusual ectopic locations [34-37]. One study of 54 self-identified Black patients with PCC/PGL in South Africa showed 39% had extra-adrenal tumors [38], and only five patients had familial syndromes; it is unclear whether these patients were assessed for all known variants [39]. Case reports have identified an *SDHB* variant in an African patient with a tumor at the aortic bifurcation, and a tumoral *HIF2α* variant in an African patient who tested negative for* VHL* and *SDHx* pathogenic variants [39,40]. While neither of our patients had tumoral tissue testing for variants, such as *HIF2α*, it is possible that they may have other acquired variants causing PGLs. There is the additional possibility of a yet-unidentified germline variant being at play, as previous genetic studies have largely involved patients of European ancestry. With the advancement in genetic testing, we may be able to identify additional pathogenic variants in diverse populations, which will serve to increase our understanding of PCC/PGL. Both patients denied a history of malignancy in relatives, and yet it is hard to say with certainty that this reflects their family history, as they admit that relatives had little access to care which likely resulted in scant information, highlighting another factor that hinders an understanding of rare disorders in individuals from disadvantaged backgrounds. Our cases raise questions that require active investigation regarding additional environmental and/or genetic factors predisposing to PCC/PGLs in so-called “rare” anatomic sites, especially in those from historically underrepresented backgrounds in medical research.

## Conclusions

As physicians, scientists, and medical educators, we are taught to value large datasets and evidence-based findings to support clinical and scientific decision-making. Yet, in the study of uncommon diseases, clinicians often rely on case reports to supply clues to pathophysiology, prognosis, and response to therapy, acknowledging limitations inherent therein. To date, understanding of diseases like PCC/PGL expanded with recent scientific discoveries; however, these have been mostly representative of subjects with European ancestry, as well as those with more access to care. For vulnerable individuals with non-European ancestries, such as those presented in our report, data for clinical, pathophysiologic, anatomic, and genetic predisposition in PCC/PGL are lacking. A scarcity of data or publications further worsens care inequities, as providers are left caring for patients with little guidance from experts in the field. Thus, we aim to improve the understanding of PCC/PGL with the expectation that this will continue to improve care and patient outcomes.
